# The Role of the Hippocampus in Passive and Active Spatial Learning

**DOI:** 10.1002/hipo.22343

**Published:** 2014-08-28

**Authors:** Yutaka Kosaki, Tzu-Ching Esther Lin, Murray R Horne, John M Pearce, Kerry E Gilroy

**Affiliations:** School of Psychology, Cardiff UniversityUnited Kingdom

**Keywords:** spatial learning, S-S versus S-R association, passive learning, length discrimination, hippocampus

## Abstract

Rats with lesions of the hippocampus or sham lesions were required in four experiments to escape from a square swimming pool by finding a submerged platform. Experiments 1 and 2 commenced with passive training in which rats were repeatedly placed on the platform in one corner—the correct corner—of a pool with distinctive walls. A test trial then revealed a strong preference for the correct corner in the sham but not the hippocampal group. Subsequent active training of being required to swim to the platform resulted in both groups acquiring a preference for the correct corner in the two experiments. In Experiments 3 and 4, rats were required to solve a discrimination between different panels pasted to the walls of the pool, by swimming to the middle of a correct panel. Hippocampal lesions prevented a discrimination being formed between panels of different lengths (Experiment 3), but not between panels showing lines of different orientations (Experiment 4); rats with sham lesions mastered both problems. It is suggested that an intact hippocampus is necessary for the formation of stimulus-goal associations that permit successful passive spatial leaning. It is further suggested that an intact hippocampus is not necessary for the formation of stimulus-response associations, except when they involve information about length or distance. © 2014 The Authors. Hippocampus Published by Wiley Periodicals, Inc.

## INTRODUCTION

Spatial learning allows animals to locate a hidden goal with reference to landmarks that are some distance from it. Evidence from a variety of sources indicates this ability is mediated by the hippocampus. To take a few examples, rats with lesions of the hippocampus find it difficult to locate a submerged platform in a swimming pool, either with reference to landmarks outside the pool (Morris et al., 1982), or with reference to the shape of the pool (Pearce et al., 2004). Alternatively, rats trained to find food in one arm of a cross maze are less likely to use spatial cues after inactivation of the hippocampus (Packard and McGaugh, 1996), and rats trained to find food in an eight-arm radial maze are similarly impaired when functioning of the hippocampus is disrupted by lesions to the fornix (McDonald and White, 1993). Although it is well established that the hippocampus plays a central role in spatial learning, the nature of this role is poorly understood. In some circumstances, spatial learning can be profoundly affected by hippocampal damage (e.g., Morris et al., 1982; Pearce et al., 2004), whereas in others this damage has rather little impact on spatial learning (e.g., White and McDonald, 1993; Jones et al., 2007). Clearly, for a complete understanding of the function of the hippocampus for successful spatial learning, it is necessary to identify the circumstances when its role in this type of behavior is major, or relatively minor.

One factor that might determine the importance of the hippocampus is the point during a spatial task at which learning takes place. Investigations of spatial learning normally require the animal to make its own way to the goal, which means that knowledge about how to find the goal in the future can be based on two rather different kinds of experience. On the one hand, there is the knowledge gained as the animal travels to the goal and, on the other hand, there is the knowledge gained when the animal is at the goal. This distinction is important because it is quite conceivable that these different circumstances result in the acquisition of different kinds of information. For example, as an animal makes its way to the goal it might learn to make a sequence of responses that are elicited by a succession of landmarks. Once the animal is at the goal, however, it may then learn about the spatial relationship between the goal and the surrounding landmarks. We consider in more detail the differences between these possibilities in the General Discussion. For the present it is sufficient to note that the former may be likened to the acquisition of instrumental stimulus-response (S-R) associations, and the latter to the acquisition of a spatial stimulus-stimulus (S-S*) association, where the initial element, S, corresponds to some or all of the stimuli surrounding the goal, and the second element, S*, corresponds to the goal itself (for recent presentations of this proposal see Sheynikhovich et al., 2009; White, 2008). There is evidence to suggest that hippocampal lesions have relatively little impact on the development of S-R associations (Packard and McGaugh, 1996) whereas, theoretically at least, they are assumed to affect adversely the formation of S-S* associations, especially when S constitutes a spatial representation, or cognitive map, of the cues surrounding the goal (O'Keefe and Nadel, 1978). On this basis, it would be expected that hippocampal lesions will disrupt more severely spatial learning that takes place at the goal, rather than on the way to the goal. One purpose of the present article is to evaluate this prediction.

In order to investigate the influence of the hippocampus on spatial learning when the animal is at the goal, the first two experiments made use of a technique that we shall refer to as passive spatial learning. In this task, rats gain experience of a goal and its relationship with surrounding cues by being repeatedly placed at the goal, rather than reaching the goal by making their own way to it. Initial attempts to use this method met with mixed success. For example, Sutherland and Linggard (1982), Keith and McVety (1988), and Jacobs et al. (1989a), all found that placing a rat on a submerged platform in a circular pool surround by landmarks aided subsequent searching for the platform when rats were released into the pool. In contrast Jacobs et al. (1989b) describe seven similar experiments which failed to find any evidence of successful passive spatial learning. As Horne et al. (2012) point out, the failures of spatial learning in these circumstances may have occurred because rats paid little attention to the relatively distant landmarks when they were on the platform. With this possibility in mind, Gilroy and Pearce (2014) and Horne et al. (2012) ensured that the relevant landmarks were salient by using a task in which they were near to the platform. In one experiment by Gilroy and Pearce, for example, rats were repeatedly placed on a submerged platform in one corner of a square pool constructed from the three white walls, and one black wall. The corner was distinctive by being constructed from the black and a white wall. After this training the rats were allowed to swim in the pool for the first time, but in the absence of the platform. Not only did rats head directly for the correct corner in preference to any other corner, but they also spent most time in this corner. Given that the rats had no experience of swimming in the pool before the test trial, the performance on the test trial confirms the effectiveness of passive spatial learning for acquiring information about where a goal is situated. With this conclusion in mind, Experiments 1 and 2 made use of the technique developed by Gilroy and Pearce to investigate the effect of hippocampal lesions on passive spatial learning. If the hippocampus is important for spatial learning when an animal is at a goal, then rats with hippocampal lesions should fail to benefit from their passive training in the square arena.

The foregoing prediction was confirmed, and replicated in Experiment 2 using a variant of the design employed for Experiment 1. Both experiments included an additional stage in which rats were required to swim to the platform on every trial, which we shall refer to as active training. The platform was situated in the same corner that was used for the passive training. If the hippocampus is not important for spatial learning that takes place as the animal makes its way to the goal, then rats with hippocampal lesions should acquire a preference for the corner housing the platform over any other corner. This prediction was confirmed and the purpose of the remaining experiments was to investigate whether there are circumstances in which even active spatial learning is severely disrupted by damage to the hippocampus.

## EXPERIMENT 1

**Figure 1:**
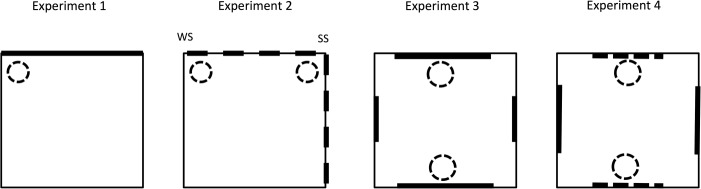
Plans of the square arena used for the four experiments. Experiment 1: Thin lines indicate white, thick lines indicate black walls. Experiment 2: Thin lines indicate white walls, dashed lines indicate striped walls. Experiment 3: Thin lines indicate gray walls, short and long thick lines signify short and long black panels. Experiment 4: Thin lines signify grey walls, thick lines signify panels with horizontal stripes, dashed lines signify panels with vertical stripes. Circles indicate possible locations for the platform. One platform was in the pool for Experiments 1 and 2, and two were in the pool for Experiments 3 and 4.

There were two groups of rats in the experiment. The hippocampal group received lesions of the hippocampus prior to the start of the experiment, and the sham group received sham lesions. The apparatus consisted of a square swimming pool with three white walls and one black wall (see left-hand panel of Fig. 1). Throughout the placement training stage, rats from both groups were repeatedly placed on a submerged platform situated in a corner created from the black wall and a white wall. They were then given a test trial in which they were allowed to swim in the pool for the first time, in the absence of the platform. The test trial was then followed by a number of sessions of active training in which rats were required to swim to the submerged platform that was situated in the corner used for placement training. The experiment concluded with a final test trial conducted in the same manner as the first test.

If a rat fell from the platform during its placement training it was discarded from the experiment. This procedure was adopted in order to ensure that no rat had experience of swimming to the platform in the presence of the cues provided by the walls of the arena. We can thus be confident that the only knowledge that rats could use when searching for the platform during their first test trial was gleaned from their experience when viewing the arena from the platform. In order to encourage rats to remain on the platform during their exposure to the square arena, three preliminary sessions of passive training were given in a circular pool.

### Materials and Methods

#### Subjects

The subjects were 24 male hooded Lister rats supplied by Harlan Olac (Bicester, Oxon, UK). They were ∼3 months old at the start of the experiment and were assigned at random to the two groups with 11 rats in the sham group. Rats were housed, in pairs where possible, in a temperature-controlled environment (∼20°C) that was continuously illuminated for 12 h per day with the lights being turned on at 07.00. Rats had free access to food and water throughout the experiment. Rats received surgery at least 2 weeks after arriving in the laboratory, and behavioral testing commenced at least 2 weeks after the completion of surgery. One rat with hippocampal lesions was removed from the experiment after gaining experience of swimming in the pool during the passive training, which resulted in the hippocampal group containing 12 rats.

#### Apparatus

The experiment was conducted in a white circular pool that was 2 m in diameter and 60 cm deep. The pool was filled to a depth of 30 cm with a mixture of water and white opacifier (500 ml, OP303B, supplied by Rohm and Haas, UK). This opaque mixture was maintained at a temperature of 25°C (±2°C) and was changed daily. A white circular ceiling with a diameter of 2 m was suspended 1 m above the top edge of the pool, and was fitted with eight, 45-W recessed spotlights. Each light was 22.5 cm in diameter. The lights were spaced evenly in a circle with diameter 1 m, concentric with the pool. In the center of the ceiling was a 30-cm hole into which a wide-angle video camera was fitted. Images from the camera were relayed to a monitor in an adjacent room, together with recording equipment, and PC with tracking software (Watermaze Software, Edinburgh, UK). This software was used to measure the amount of time spent in different areas of the pool.

Three white and one black polyurethane boards could be inserted into the pool to create the square-shaped arena. They were 141 cm in length, 60 cm high, and 4 mm thick. A gray curtain was drawn around the pool throughout the experiment to mask any extra-maze visual cues. It was hung at a distance of 25 cm beyond the edge of the pool, and covered the entire height from the ceiling to below the pool's edge.

A circular clear-Perspex platform with a diameter of 10 cm was placed in one of the corners created by a white wall and the black wall. The center of the platform was situated on a notional line that bisected the corner, at a distance of 25 cm from the corner. The platform was mounted on a column so that its upper surface was 2 cm below the surface of the water.

#### Surgery

The rats were anaesthetized with a mixture of isoflurane (1–5%) and oxygen, and placed in a stereotaxic frame (David Kopf Instruments, Tujunga, CA). The incisor bar was set at −3.3 mm. The scalp was incised at the midline to expose the skull. A dental drill was used to remove the skull over the region to be lesioned. A 2-µl Hamilton syringe was used to infuse 63-mM ibotenic acid (Tocris Bioscience, Bristol, UK) dissolved in phosphate-buffered saline (pH 7.4) bilaterally into the hippocampus. The coordinates were the same as those used by Jarrard (1989). The infusion was made with an infusion pump at the rate of 0.03 µl/min, and each infusion was followed by a 2-min diffusion period during which the needle was left in place.

After the infusions had been completed, the wound was sutured and the rats were allowed to recover in a warm chamber until conscious. A 5-ml mixture of glucose and saline was injected subcutaneously after surgery to aid recovery, and antibiotic powder (Aureomycin, Fort Dodge, Animal Health, Southampton, UK) was topically applied. Rats were also given the analgesic Metacam (0.06 ml s.c.; 5 mg/ml meloxicam; Boehringer Ingelheim Vetmedica, Germany). Sham-operated rats underwent the same surgical procedure except that the Hamilton syringe was not lowered into the brain, and that the dura was perforated with the tip of a 25-gauge needle on three sites per hemisphere. Following a minimum of 14 days of postoperative recovery period, the behavioral testing began.

#### Histology

After the completion of the present experiment, all the rats participated in Experiment 3. Upon the completion of that study, the animals were administered a lethal dose of sodium pentobarbitone (Euthatal). They were then transcardially perfused with 0.9% saline, and then with 10.0% formal-saline. The brains were postfixed for 24 h, and then placed in phosphate-buffered (0.1 M) 30.0% sucrose solution for a further 24 h. The brains were then frozen in a −20°C cryostat before they were sliced along the coronal plane creating 40-µm sections. These were placed on gelatine-coated slides which were left to dry for 24 h at room temperature. After they had been dried they were stained with cresyl violet and examined under a microscope, and the extent of the damage to the hippocampus was established using the boundaries defined by Paxinos and Watson (2005).

#### Procedure

Rats were trained in groups of five or six. For 5 days a week, the rats were carried to the room adjacent to the test room in an aluminum, light-tight box with individual compartments. After each of the four trials of a session a rat was dried with a towel before being returned to the box. The remaining rats in the box were treated in the same way before the original rat was removed for its subsequent trial. The inter-trial interval (ITI) was ∼5 min.

Pretraining took place in the circular pool in the absence of the black and white boards, and with the curtain drawn around the pool. Rats were placed on the platform and expected to remain on it for 30 s. Any rat that left the platform was guided back by placing a finger in front of its snout and moving the finger slowly towards the platform. On returning to the platform, the rat was allowed to remain on it for the remainder of the 30 s. By the third, and final, session of pretraining, all the rats remained on the platform for the full 30 s. There were eight possible positions for the platform, which were located on notional lines that bisected each of the four quadrants of the pool, at a distance of either 25 cm or 50 cm from the edge of the pool. The position of the platform was randomized between trials with the stipulation that each quadrant was used once in each session and that the platform was 25 cm from the edge on two of the trials and 50 cm from the edge on the remaining two trials.

The eight sessions of passive training took place in the square pool with three white walls and one black wall. Each rat was repeatedly placed on the platform for 30 s, facing a corner created by the black and a white wall. For five rats in each group the platform was always situated in a corner where the black wall was to the left of a white wall, and for the remaining rats the platform was situated in the corner where the black wall was to the right of a white wall. The arena was rotated within the pool by 90, 180, or 270° from one trial to the next in a random sequence, with the constraint that any given corner occupied four different locations, with reference to the experimental room, within each session. The fourth trial of Session 8 was a test, conducted in the absence of the platform, in which rats were allowed to swim in the pool for 60 s. With reference to the experimental room, there were four possible orientations of the arena, and four possible positions for the experimenter to stand when releasing a rat for the test trial. These factors were varied randomly for each rat. The rats were released into the center of the pool, facing the experimenter. The experimenter always stood beside the center of a wall when releasing the rat, and then moved to the adjoining room to observe the rat on the monitor.

The next four sessions involved active training with rats being required to swim to the platform, which was situated in the same place as for passive training. For each of the four trials in a session, rats were released from the centre of one of the walls, facing the wall, and were given 60 s to find the platform. If the animal did not find the platform in the allotted time, then they were guided to it by the experimenter. The wall from which the animal was released was randomized between trials with the restriction that each wall was used once in a session. The orientation of the arena in relation to the room was also randomized so that each 90° rotation was used once in a session. On reaching the platform, the rat was allowed to remain on it for 30 s. The experiment concluded with a test trial, which took place on the fourth trial of the final session of active training, and which was conducted in the same manner as the original test trial.

### Data Analysis

The behavior of every rat was observed on the monitor connected to the camera throughout the experiment. During a test session, the rat's movements were tracked on the computer, using Watermaze software (Morris and Spooner, 1990). For the purposes of analyzing the results from the test trials, circular search zones in each corner were used. The zones had a diameter of 30 cm with their centers located at a distance of 25 cm from the corner, equidistant from the walls creating the corner. The software recorded the percentages of the two 60-s test trials that were spent in each zone.

## RESULTS

### Histology

**Figure 2:**
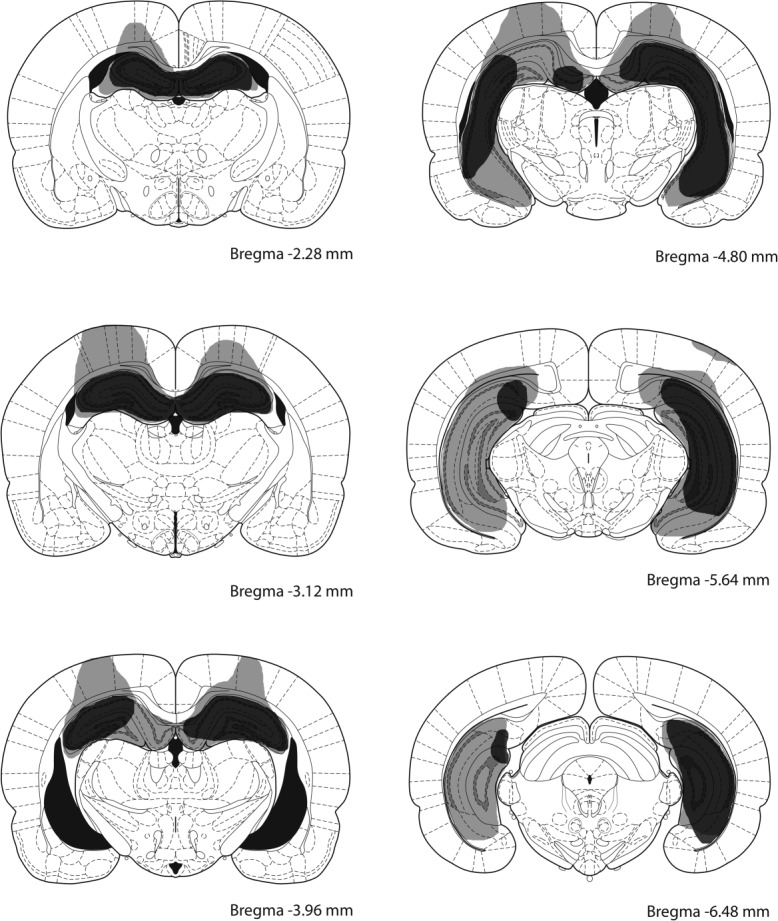
Schematic representation of ibotenic acid lesions of the hippocampus in rats for Experiment 1 and 3. The largest and smallest extents of neuronal damage are represented in light gray and dark grey, respectively. Atlas plates are adapted from Paxinos and Watson (2005).

Figure 2 shows the extent of damage to the hippocampus induced by the injections of ibotenic acid. We employed a criterion for successful lesion of more than 50% cell loss at the dorsal hippocampus (at the level between 2.28 and 3.96 mm posterior to Bregma, shown in the first three panels from the top of Fig. 2). On the basis of this criterion, three animals exhibited insufficient lesions and therefore were removed from subsequent analyses. In one rat damage to the overlying cortical area was too large and the subject was also removed. The lesion for the remaining eight hippocampal animals typically produced almost complete loss of cells in the dorsal hippocampus while some variation was observed in the extent to which ventral and posterior part of the hippocampus was damaged, as shown in the figure.

### Behavior

**Figure 3:**
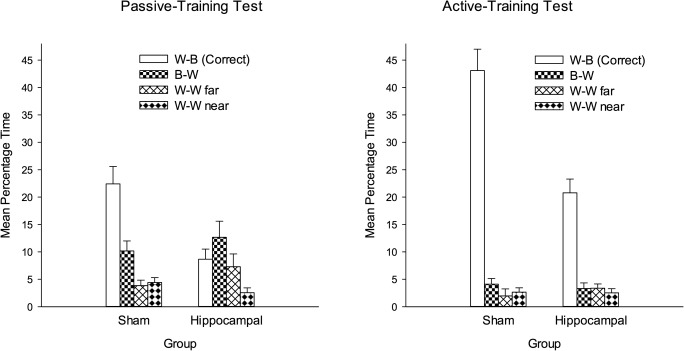
The mean percentage of time spent by the two groups in the four corners of the square pool during the test trial after passive training (left-hand panel) and active training (right-hand panel) of Experiment 1. W, white wall; B, black wall. Error bars show the standard error.

The left-hand panel of Figure 3 shows the mean percentages of time spent by the two groups in the four corners of the square arena during the test trial after the completion of passive training. For the purposes of discussion, the data have been normalized so that the corner where the platform was situated—the correct corner—is referred to as corner W-B, where the white (W) wall was to the left of the black (B) wall. The remaining corners are referred to as B-W, W-W far (diagonally opposite the correct corner), and W-W near (adjacent to the correct corner).

The results show clearly that the passive training was effective in the sham group, with considerably more time being spent in the correct corner than any other corner. In contrast, not only did the hippocampal group spend substantially less time in the correct corner than the sham group, but it also failed to express a clear preference for the correct corner over the three remaining corners. It thus appears that the effects of passive training were disrupted by lesions of the hippocampus. For the purposes of statistical analysis, the time spent in the correct corner was compared with the mean time spent in the remaining three corners. A two-way ANOVA of these data revealed significant effects of lesion, *F*(1, 17) = 14.79, *P* < 0.001, *MSE* = 24.05, and corner, *F*(1, 17) = 10.90, *P* < 0.01, *MSE* = 64.74, and a significant interaction, *F*(1, 17) = 8.17, *P* < 0.05, *MSE* = 64.74. Simple main effects tests then indicated that the time spent in the correct corner was significantly greater than the mean time spent in the remaining three corners for the sham group, *F*(1, 17) = 18.96, *P* < 0.001, *MSE* = 64.74, but not the hippocampal group, *F* < 1. In addition, significantly more time was spent in the correct corner by the sham than the hippocampal group, *F*(1, 34) = 19.73, *P* < 0.001, *MSE* = 44.37, but the equivalent difference for the remaining three corners was not significant, *F* < 1.

Although the foregoing pattern of results indicates that passive spatial learning is disrupted by lesions of the hippocampus, one finding is at odds with this conclusion. Figure 3 shows that the hippocampal group spent more time in the correct corner during the test trial than in the nearer of the two white corners. Moreover, this difference was statistically significant, *t*(7) = 3.17, *P* < 0.05. A possible explanation for this outcome is that the hippocampal group gained some benefit from the placement training it received. This group might, for example, have learned that the platform was situated close to a black wall, but not appreciated which of the two corners at the end of the wall was correct. Another possibility is that the results reflect the influence of nothing more than an unconditioned preference for searching in dark, rather than bright corners. In keeping with this possibility, it can be noted that the sham group spent significantly more time in the incorrect black and white corner than either of the white corners during the test trial, *t*s(10) > 2.07, *Ps* < 0.05. Such an outcome would be expected if rats have a natural aversion to bright corners in our test arena. We shall return to this matter shortly. In the meantime, the results thus far demonstrate for the first time a disruptive effect of hippocampal lesions on passive spatial learning in a square arena with distinctive walls.

**Figure 4:**
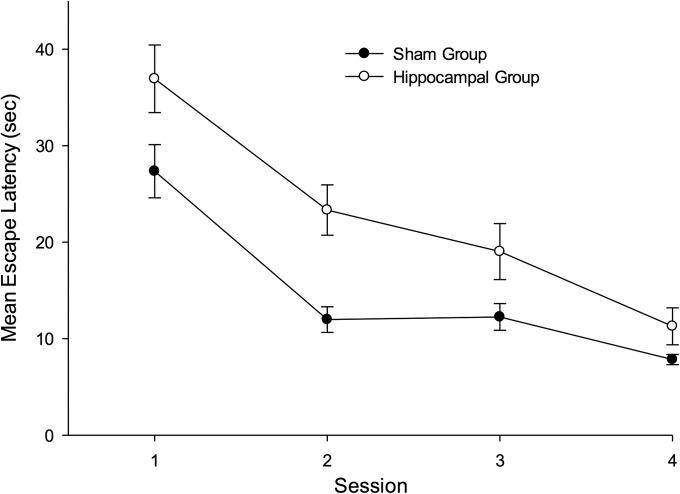
Mean escape latencies for the two groups during the four sessions of active training in Experiment 1. Error bars show the standard error.

In order to reveal the effects of hippocampal damage on active spatial learning, the experiment included four sessions of training in which rats were required to swim to the platform. Figure 4 shows the mean escape latencies for the two groups for each session of this training. There was a marked reduction in the escape latencies of both groups as training progressed, but the performance of the sham group was consistently superior to that of the hippocampal group. A two-way ANOVA revealed a significant effect of group, *F*(1, 17) = 21.69, *P* < 0.001, MSE = 51.84, and a significant effect of session, *F*(3, 51) = 40.46, *P* < 0.001, *MSE* = 41.95, but the interaction was not significant, *F*(3, 51) = 1.32, *P* > 0.25.

The results from the test trial at the end of active training are shown in the right-hand panel of Figure 3. Both groups spent substantially more time in the correct corner than any other corner, although the magnitude of this effect was more marked in the sham than the hippocampal group. The time spent in each of the three remaining corners was small for both groups. A two-way ANOVA similar to the one described above revealed a significant effect of lesion, *F*(1, 17) = 20.67, *P* < 0.001, MSE = 55.09, of corner, *F*(1, 17) = 117.10, *P* < 0.001, MSE = 66.39, and a significant interaction, *F*(1, 17) = 17.55, *P* < 0.001, MSE = 66.39. Subsequent tests of simple main effects revealed a significant effect of corner in the hippocampal, *F*(1, 17) = 21.96, *P* < 0.001, MSE = 66.39, and the sham group, *F*(1, 17) = 112.60, *P* < 0.001, MSE = 66.39. They also revealed a significant difference between the groups in the time spent in the correct corner, *F*(1, 34) = 37.94, *P* < 0.001, MSE = 60.74, but not in the other corners, *F* < 1.

In contrast to the effects of the passive training, being required to swim to the platform resulted in the hippocampal group acquiring a stronger preference for the correct corner over any other corner. It thus appears that damage to the hippocampus exerts a more profound influence on passive rather than active spatial learning. Some comment is needed concerning the finding that the hippocampal group spent less time in the correct corner than the sham group during the final test trial. One explanation for this outcome is that the effects of active training were disrupted to some extent by hippocampal lesions. An alternative explanation is that the effects of the active training were the same in both groups, and the difference between them was due solely to the sham group being able to take advantage of its experiences on the platform for finding the goal. A measure of support for this second alternative is provided by the finding that both groups spent a very small amount of time in the wrong corners during the test trial. Such an outcome might not be expected if both active and passive spatial learning were impaired in the hippocampal group.

## EXPERIMENT 2

A potential problem with Experiment 1 was the use of a black wall in order to create a distinctive corner in an otherwise white arena. It was suggested that the results of the test to investigate the effects of the placement training may have been compromised by an unconditioned preference for rats to seek a dark rather than a bright corner. Support for this suggestion can be found in an experiment by Gilroy and Pearce (2014) in which rats received placement training similar to that used in Experiment 1, but in an arena with two adjacent black walls, and two adjacent white walls. Even though passive training took place in a corner created by a black and a white wall, during the test trial rats chose to spend the bulk of their time in the corner created by two black walls. This preference was weakened substantially in a related experiment in which the rats were trained and tested in a pool with two adjacent white walls, and two adjacent black and white striped walls, each with a white stripe at either end. After receiving passive training in a corner with a white wall and a striped wall, during a subsequent test trial rats expressed a strong preference for the correct corner and rather little interest in the corner created by two striped walls. In view of this finding, the effects of passive training were examined again in Experiment 2, but this time with striped rather than black walls. Training took place in a pool with two adjacent white walls, and two adjacent striped walls (see Fig. 1) and there were again rats with hippocampal lesions and rats with sham lesions. For rats in the hippocampal-WS and the sham-WS groups, the platform was situated in a corner created by two different walls – one white, and one striped. On the basis of the previous experiment, it was expected that the hippocampal-WS group would spend considerably less time than the sham-WS group searching in the correct corner during the test trial. Of perhaps more interest was the question of whether the hippocampal-WS group would again exhibit a preference for the correct corner over one or more of the other corners. Evidence of such a preference would suggest that the hippocampal lesions did not disrupt entirely the effects of passive training. Moreover, given the change in apparatus, it would be harder than in Experiment 1 to explain this outcome in terms of rats expressing an unconditioned preference for the correct corner over at least one of the other corners. Of course, if rats were to treat all four corners equally, then it would imply that the lesions had a profound impact on passive spatial learning.

An additional two groups were included in the experiment in order to gain an insight into any disruptive effect the lesions might have on performance during the test trial. According to several authors, the hippocampus is important for constructing representations of complex objects, and the spatial relations among the components of those objects (Rudy and Sutherland, 1989; Aggleton and Pearce, 2001; O'Reilly and Rudy, 2000,^2001^). According to this kind of proposal, the lesions might be effective by making it difficult for animals to differentiate between a corner where a striped wall is to the left of a white wall, and the mirror image of this corner. The remaining two groups in the experiment, hippocampal-SS and sham-SS, were included in the experiment in order to test the foregoing possibility. Passive training took place in the arena just described, but the platform was located in the corner created by two striped walls. In order to differentiate between this corner and the remaining three in the arena, there is no need for animals to refer to the spatial relationship between the walls creating the corner. All that is necessary is to seek a corner with only striped walls. If hippocampal lesions make it hard to appreciate the spatial relationship between stimuli, then during the test trial, the hippocampal-SS group should be able to identify the correct corner, but the hippocampal-WS group should not. In keeping with Experiment 1, the initial test trial was followed by a number of sessions of active training, with the platform in the same location as for passive training, followed by a final test trial.

### Materials and Methods

#### Subjects

The 48 male Lister-hooded rats were from the same stock and maintained in the same way as for the previous experiments. Half the rats received hippocampal lesions, and half were sham-operated, in the same manner as for Experiment 1. At least 14 days after the completion of the surgery the rats received trace conditioning with visual and auditory stimuli and foot shock. A further 14 days elapsed between the end of trace conditioning and the start of the present experiment, at which point rats were assigned to the four groups: hippocampal-WS, sham-WS, hippocampal-SS and sham-SS. Two rats were removed from the experiment because they left the platform during placement training. One swam in the pool and the other repeatedly jumped onto the walls of the arena, leaving 12 animals in the hippocampal-WS and the sham-SS groups, and 11 in the two remaining groups.

#### Apparatus

The apparatus was the same as that used for Experiment 1, with the addition of two striped walls. The striped walls were made by attaching seven vertical strips of black plastic adhesive film (Deco d-c-fix) to white walls. The stripes were 10 cm wide, separated by a gap of 10 cm, and extended from the top of the boards to below the surface of the water. There was a white stripe of between 5 and 6 cm width at each edge of the striped walls.

#### Surgery

The surgical procedure was the same as for Experiment 1.

#### Procedure

All aspects of the pretraining, training, and test were the same as for Experiment 1, except that the corner where the platform was situated was created by a striped and a white wall for the sham-WS and the hippocampal-WS groups, and by two striped walls for the sham-SS and the hippocampal-SS groups. There were eight sessions of placement training, with four trials in each session. The test trial was conducted during the fourth trial of Session 8. For each placement trial the rat was placed on the platform for 30 s before being removed from the pool. Following the completion of the first test trial there were seven sessions of active training, with four trials in each session. The manner of this training was the same as for Experiment 1. On the final trial of the seventh session, the animals were given a single test trial, which was identical to the test trial given in the previous experiment.

## RESULTS

### Histology

**Figure 5:**
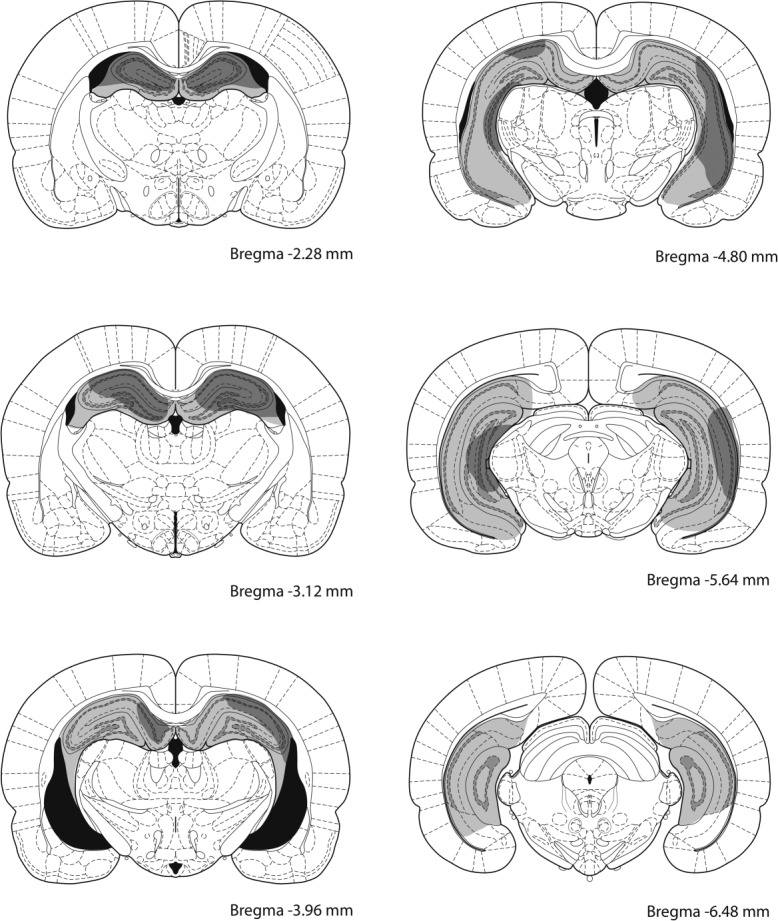
Schematic representation of ibotenic acid lesions of the hippocampus in Experiment 2. The largest and smallest extents of neuronal damage are represented in light grey and dark gray, respectively. Atlas plates are adapted from Paxinos and Watson (2005).

Figure 5 shows the extent of damage to the hippocampus induced by the injections of ibotenic acid. We employed a criterion for successful lesion of more than 50% cell loss at the dorsal hippocampus (at the level between 2.28 and 3.96 mm posterior to Bregma, shown in the first three panels from the top of Fig. 5). On the basis of this criterion, one rat was removed from hippocampal-SS group. Of the remaining 22 hippocampal rats that were included for subsequent behavioral analyses, 20 rats sustained more than 90% damage to the dorsal hippocampus, while some variation was observed in the extent to which ventral hippocampus was damaged, as shown in the figure.

### Behavior

**Figure 6:**
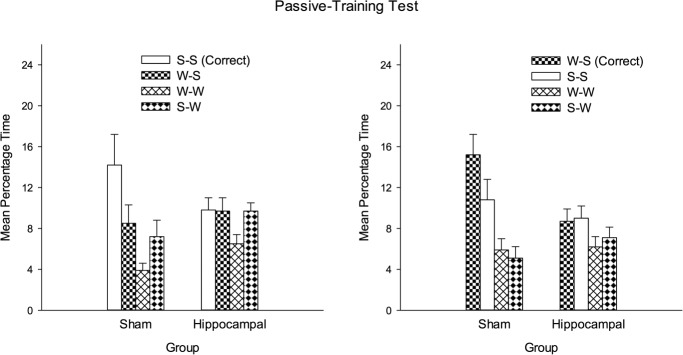
The mean percentages of time spent by different pairs of sham and hippocampal groups in Experiment 2 after passive training with the platform in a corner of the square pool created by two striped walls (left-hand panel), or in a corner created by a white and a striped wall (right-hand panel). S, striped wall; W, white wall. Error bars show the standard error.

The mean time spent in each corner of the arena by the four groups during the first test trial, which took place after the passive training, can be seen in Figure 6. The results for the sham-SS and hippocampal-SS groups are shown in the left-hand panel, and the results from the sham-WS and hippocampal-WS groups are shown in the right-hand panel. For each group, the mean time spent in the correct corner is depicted by left-hand bar of each set of four bars. For the purposes of presentation, the results of the sham-WS and hippocampal-WS groups have been normalized so that the corner where the white wall was to the left of the striped wall (W-S) is regarded as the correct corner. A comparison of the results in the left-and right-hand panels of the figure reveals that the position of the platform during training had little impact of the outcome of the test trial. In addition, both hippocampal groups failed to express a clear preference for one corner over the others, whereas both sham groups spent more time in the correct corner than any other corner. In order to analyze the results from the four groups the time spent in the correct corner was compared with the mean time spent in the remaining three corners. A three-way ANOVA revealed a significant effect of lesion, *F*(1, 41) = 8.47, *P* < 0.01, *MSE* = 18.71, of corner, *F*(1, 41) = 13.42, *P* < 0.001, *MSE* = 33.36, and a significant Lesion × Corner interaction, *F*(1, 41) = 9.50, *P* < 0.01, *MSE* = 33.36. The between-group effect of location (whether the platform was situated in Corner SS or WS), and all the interactions involving this factor were not significant, *F*s < 1. Investigation of the Lesion × Corner interaction revealed a significant effect of corner for the two sham groups combined, *F*(1, 41) = 22.75, *P* < 0.001, *MSE* = 33.36, but not for the two hippocampal groups combined, *F* < 1. In addition, significantly more time was spent in the correct corner by the two sham groups combined, than the two hippocampal groups, *F*(1, 82) = 17.39, *P* < 0.001, *MSE* = 26.04. The equivalent comparison for the mean time spent in the remaining three corners was not significant, *F* < 1.

The result obtained in both arenas correspond closely with the findings from Experiment 1, by showing that hippocampal lesions disrupt severely passive spatial learning. Moreover, the disruptive influence was unaffected by whether the platform was situated in a corner created either by two striped walls, or by a striped wall in a particular spatial relation with a white wall. This finding strongly suggests that the lesions are not effective because they make it difficult for rats to appreciate the spatial relationship between two or more objects. If this were the case then passive training should have been effective in the hippocampal-SS group.

**Figure 7:**
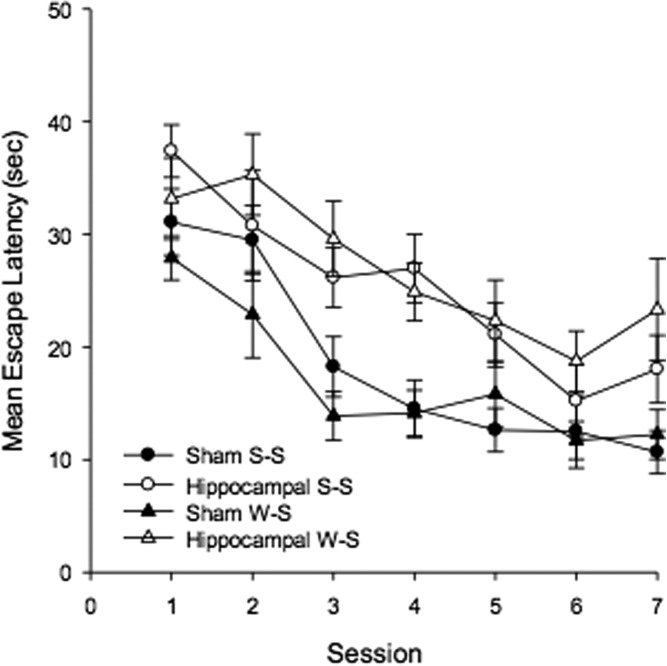
The mean escape latencies for the four groups during active training in Experiment 2. Error bars show the standard error.

The mean time taken to reach the platform by each group during active training can be seen in Figure 7. There was a consistent reduction in these times as training progressed but, throughout this stage, the performance of the groups with sham lesions was superior to the groups with hippocampal lesions. A three-way ANOVA of individual mean times to reach the platform for the four groups revealed a significant effect of lesion *F*(1, 41) = 21.09, *P* < 0.01, MSE = 217.65, and of session, *F*(6, 246) = 25.71, *P* < 0.01, MSE = 79.52, but the effect of corner (W-S or S-S) was not significant, *F* < 1. The Lesion × Corner interaction was not significant, *F* < 1, and neither were the remaining interaction, *Fs*(6, 246) < 1.01, *Ps* > .48, MSE = 79.34.

**Figure 8:**
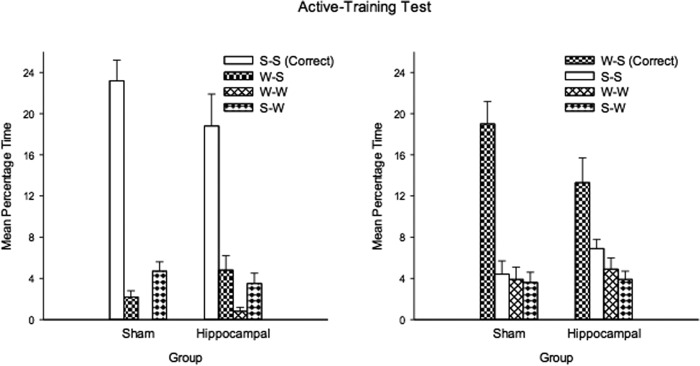
The mean percentages of time spent by different pairs of sham and hippocampal groups in Experiment 2 after active training with the platform in a corner of the square pool created by two striped walls (left-hand panel), or in a corner created by a white and a striped wall (right-hand panel). S, striped wall; W, white wall. Error bars show the standard error.

The presentation of the results from the test trial that was conducted after the completion of active training (see Fig. 8), and the manner in which they were analyzed, is the same as for the first test trial. The striking difference between the results shown in Figures 8 and 6, is that active training resulted in the two hippocampal groups acquiring a strong preference for the correct corner. The magnitude of this preference was not as great as for the sham groups. There is also an indication that the sham-SS and the hippocampal-SS groups spent more time in the correct corner than their counterparts trained to find the platform in a corner with a white wall and a striped wall. These observations were supported by an ANOVA, which was performed in the same way as for the first test trial. The effects of lesion, *F*(1, 41) = 6.25, *P* < 0.05, *MSE* = 23.18, of corner, *F*(1, 41) = 130.08, *P* < 0.001, *MSE* = 36.27, and the Lesion × Corner interaction, *F*(1, 41) = 7.61, *P* < 0.01, *MSE* = 36.27, were all significant. The Location × Corner interaction was also significant, *F*(1, 41) = 5.38, *P* < 0.05, *MSE* = 36.27. Tests of simple main effects, to examine the significant Lesion × Corner interaction, confirmed that the time spent in the correct corner was significantly greater than the mean of the time spent in the remaining three corners for the two hippocampal groups, *F*(1, 41) = 37.38, *P* < 0.001, *MSE* = 36.27, and for the two sham groups, *F*(1, 41) = 100.31, *P* < 0.001, *MSE* = 36.27. Moreover, the sham groups spent significantly more time than the hippocampal groups in the correct corner, *F*(1, 82) = 13.80, *P* < 0.001, MSE = 29.69, but the time spent by the sham groups and the hippocampal groups in the three remaining corners did not differ significantly, *F* < 1. Examination of the Location × Corner interaction indicated that significantly more time was spent in the correct corner when it was made from two striped walls, than when it was made from a white wall and a striped wall, *F*(1, 82) = 5.56, *P* < 0.05, MSE = 29.69. To return to the three-way ANOVA, the effect of location, and the remaining interactions involving this factor were not significant, *F*s < 1.

The results from the second test trial confirmed the finding from Experiment 1 that damage to the hippocampus does not eliminate the effectiveness of active training in our experimental environment. In keeping with Experiment 1, the experiment also revealed that during the test trial the sham groups spent more time than the hippocampal groups in the correct corner of the arena. As noted earlier, it is hard to say whether this difference was due to the lesions having some disruptive effect on active spatial learning, or whether it was due to the sham groups, but not the hippocampal groups, benefitting from the effects of passive spatial learning.

## EXPERIMENT 3

The experiments thus far indicate that hippocampal lesions have relatively little impact on active spatial learning. It would, however, be premature to conclude that all aspects of active spatial learning are relatively immune to the effects of hippocampal damage. A variety of experiments have required animals to make their own way to the goal. While some, like Experiments 1 and 2, failed to find a substantial impact of hippocampal lesions on these tasks, others have revealed a profound disruption on the ability to locate the goal. (e.g. Morris et al., 1982, McDonald & White, 1993; Pearce et al., 2004). These contrasting findings imply that hippocampal lesions disrupt active spatial learning in some circumstances, but not others, which raises the question of under what conditions of active training are the lesions effective. We noted in the Introduction that active spatial training is likely to be effective because it enables animals to find the goal by responding in a particular manner with reference to stimuli in the vicinity of the goal. That is, it permits the development of S-R habits. It is unlikely that the lesions are effective because they disrupt the formation of S-R associations in general, because there is good evidence of effective S-R learning in rats when activity in the hippocampus is severely disrupted (see Morris et al., 1982; McDonald & White, 1993; Packard & McGaugh, 1996). An alternative possibility is that hippocampal lesions are effective by affecting the selection of stimuli that animals rely on when heading for a goal. In the case of Experiments 1 and 2, it would seem that hippocampal lesions do not impair the capacity to use white walls, black walls, and striped walls as cues for finding the goal, but it is possible that they might impair the capacity to use other kinds of cue. If this analysis is correct then, in spite of the failures to reveal a severely disruptive effect of hippocampal lesions on active spatial learning in Experiments 1 and 2, it should be possible to reveal a greater influence in our test environment by selecting a suitable stimulus for indicating where the platform can be found.

With this rationale in mind, Experiment 3 examined the impact of hippocampal lesions on the ability of rats to solve a discrimination based on the length of objects. Pearce et al. (2004) reported that rats with hippocampal lesions are unable to identify the corner in which a platform is situated in a rectangular pool with four walls of the same colour. They therefore suggested that the lesions prevented animals from being able to differentiate between the long and the short walls of the arena (McGregor et al., 2004; Jones et al., 2007; Pearce, 2009). If this suggestion is correct, then it should be possible to demonstrate an impairment of active spatial learning in a similar environment to that used for Experiments 1 and 2, when the location of the goal is indicated by objects of different lengths. In order to test this prediction, the next experiment was based on a design developed by Kosaki et al. (2013). A sham and a hippocampal group were required to escape from a square pool with four grey walls to each of which was attached a single black panel. The panels were of the same height but of two different widths (see Fig. 1). Submerged escape platforms were situated beside the middle of the long but not the short panels in both groups. Kosaki et al. demonstrated that this methodology will encourage a preference for searching near the long rather than the short panel. If the hippocampus is important for making discriminations based on the length of objects, then the hippocampal group should fail to acquire this preference. The experiment was conducted in two phases, in the first phase the lengths of the panels were 100 cm and 50 cm, and it was found that rat with hippocampal lesions were unable to search preferentially for the platform in front of the long panel. In view of this outcome the task was simplified for the second phase of training by using panels that were either 100 cm or 25 cm wide. Despite this change the hippocampal group again failed to acquire a preference for the long over the short panels. While this outcome is consistent with the claim that the hippocampus disrupts active spatial learning, when it is based on the length of objects, a more prosaic explanation is possible for our results. The lesions may have made it difficult for rats to solve any discrimination when the cues are pasted to the walls of a square pool. The final experiment that we report was designed to test this possibility.

In keeping with the design used by Kosaki et al., a landmark was attached at the top of the walls, to the center of each of them. The landmarks were identical and were thus of no help for distinguishing between the long and short panels, but they could be used as an aid for finding the center of a panel.

### Materials and Methods

#### Subjects

The rats were the same as those used for Experiment 1 and the experiment commenced approximately two weeks after the completion of Experiment 1. The manner of housing was the same as for Experiment 1.

#### Apparatus

The experiment was conducted in the same pool as Experiment 1, in a square arena constructed from four polyurethane gray walls with the same dimensions as for Experiment 1. A panel of black plastic self-adhesive vinyl film with a height of 45 cm and a width of 100 cm was attached to the middle of two opposing walls. The two other walls were similarly covered by a panel of the same material cut to a width of either 50 or 25 cm. The centers of the panels were superimposed on a notional vertical line passing through the center of the wall (see Fig. 1). The bottom of the black panels extended below the water surface, and the top was 2 cm below the top edge of the wall. As for Experiment 1, a gray curtain was drawn around the pool throughout the experiment to exclude any extra-maze cues. Two identical platforms were used for the experiment. They were made from the same material and of the same dimensions as the platform described in Experiment 1. Each platform was positioned with its center 15 cm from the midpoint of one of the two 100-cm black panels, on a notional line that was perpendicular to the wall.

Four identical balls, 10 cm in diameter and covered in colored cartoon characters, were used as landmarks. They were supported by clear Perspex, horizontal rods attached to the middle of the top of each wall. The centers of the landmarks were positioned 15 cm away from the wall to which they were attached. When a landmark was above a platform, its center was directly above the center of the platform.

#### Procedure

Details of the surgery can be found in Experiment 1. During Phase 1 of the experiment there were four training trials in each of 10 sessions. A training trial started with a rat being released gently into the pool facing a corner. Rats were released from each of the four corners once in a session, in a randomly selected sequence. Following 10 sessions of training, a single 60-s test trial was conducted on Day 11. The panels attached to the walls throughout this phase were 100 and 50 cm wide. The treatment for Phase 2 was identical to Phase 1, except that the widths of the panels were 100 and 25 cm, and there were two test trials, one after the fifth session of training and one after the tenth. For every training trial the time taken to reach a platform after being released into the pool was recorded. During each test trial, a record was taken of the amount of time that was spent in four circular search zones of diameter 30 cm. The centers of the zones were directly beneath the centers of the landmarks. A record was also taken of which search zone a rat entered first. Procedural details that have been omitted were the same as for Experiment 1.

## RESULTS

**Figure 9:**
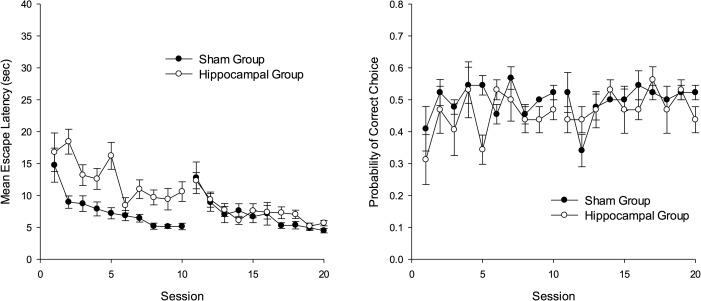
The mean escape latencies and the mean probabilities of making a correct choice for the two groups during the 10 sessions of Phase 1, and the 10 sessions of Phase 2 of Experiment 3. Error bars show the standard error.

Details of the histology are described in Experiment 1. The left half of the left-hand panel of Figure 9 show the group mean escape latencies for the two groups for each session of training in Phase 1. The time taken to reach the platform was consistently faster for the sham than the hippocampal group. In support of this observation, a two-way ANOVA revealed a significant effect of lesion, *F*(1,17) = 21.15, *P* < 0.001, *MSE* = 54.93. There was also an effect of session, *F*(9,153) = 10.30, *P* < 0.001, MSE = 15.40, indicating a reduction in the escape latencies as training progressed, but no Lesion × Session interaction, *F*(9,153) = 1.94, *P* > 0.05, MSE = 15.40.

The left half of the right-hand panel of Figure 9 shows the mean probability of choosing a correct search zone immediately after being released into the pool for the 10 sessions of training in Phase 1. Neither group showed much improvement in this measure as training progresses, although there is a hint that the performance of the sham group was superior to that of the hippocampal group. A two-way ANOVA of individual mean probabilities for each of the 10 sessions revealed a significant effect of lesion, *F*(1, 17) = 6.70, *P* < 0.05, MSE = 0.022, and of session, *F*(9, 153) = 2.30, *P* < 0.05, MSE = 0.022, but the interaction was not significant, *F*(9, 153) = 1.03, *P* > 0.10. One sample *t-*tests, in which individual mean probabilities of selecting a correct zone before an incorrect zone, for the final five sessions combined, were compared with a chance level of 0.5 failed to reveal a significant effect for the sham, *t*(10) = 0.0, *P* > 0.10, or the hippocampal, *t*(7) = 0.93, *P* > 0.10, group.

**Figure 10:**
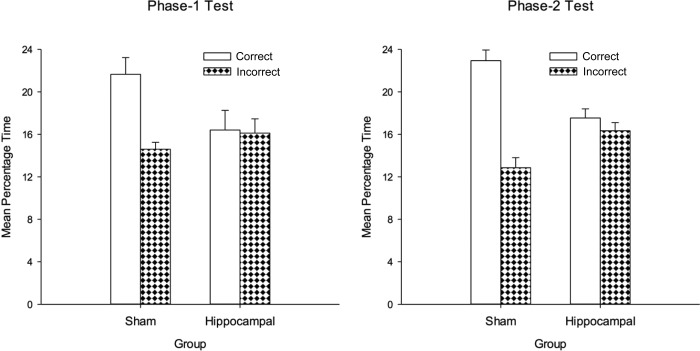
The mean percentages of time spent in the correct and incorrect search zones during the test trials at the end of Phase 1 (left-hand panel) and Phase 2 (right-hand panel) for the sham and hippocampal groups of Experiment 3. Error bars show the standard error.

The mean durations of the time spent by the two groups in the correct and incorrect search zones for the first test trial are displayed in the left-hand panel of Figure 10. During this test the sham group spent more time in the two correct search zones than in the two incorrect search zones; the hippocampal group spent virtually the same amount of time in both kinds of zone. A two-way ANOVA revealed a significant Lesion × Zone interaction, *F*(1,17) = 5.56, *P* < 0.05, MSE = 19.04. There was also a significant effect of zone, *F*(1,17) = 6.54, *P* < 0.05, MSE = 19.04, but not of lesion, *F*(1,17) = 1.87, *P* > 0.10, MSE = 17.17. Simple main effects analysis of the interaction showed that the sham group spent significantly more time in the correct than incorrect zones, *F*(1,17) = 14.34, *P* < 0.01, *MSE* = 19.04, whereas the hippocampal group did not, *F* < 1. In addition, the sham group spent more time in the correct zones than the hippocampal group, *F*(1,17) = 4.62, *P* < 0.05, *MSE* = 27.53, but there was no difference between groups in the time spent in the incorrect zones, *F*(1,17) = 1.23, *P* > .10.

The right-hand half of each panel in Figure 9 shows the performance of the groups during the 10 sessions of training in Phase 2. Replacing the discrimination between panels that were 100 cm and 50 cm wide, with one where the widths were 100 cm and 25 cm, improved the performance of the hippocampal group, whose mean escape latencies were similar to those of the sham group. A Lesion × Session ANOVA revealed a significant effect of session, *F*(9, 153) = 9.88, *P* < 0.001, *MSE* = 9.03. There was no effect of lesion or Lesion × Session interaction, *Fs* < 1.

The probability of swimming directly to a correct zone after being released was also very similar for each group during Phase 2, and showed only a slight improvement as training progressed. The effect of session, *F*(9, 153) = 1.94, *P* = .05, MSE = 0.017, was significant, but the effect of lesion, *F* < 1, and the interaction, *F* < 1, were not significant. In order to determine if Phase-2 training resulted in rats preferentially heading for a correct rather than incorrect zone on being released into the pool, one sample t-tests similar to those performed for the first test trial were conducted. These tests failed to reveal a significant effect for the sham, *t*(10) = 2.19, *P* = 0.053, or the hippocampal, *t*(7) = 1.36, *P* > 0.10, group.

The results from the test trial after Sessions 5 and 10 of Phase 2 were similar, and therefore combined for the sake of ease of presentation. The right-hand half of Figure 10 shows a very similar pattern of results to that found in the first test trial with the hippocampal group again failing to discriminate between the short and long panels. A similar ANOVA to the one conducted for the first test trial revealed a significant effect of zone, *F*(1, 17) = 26.59, *P* < 0.001, and Lesion × Zone interaction, *F*(1, 17) = 16.49, *P* < 0.01, MSE = 11.07. Subsequent tests of simple main effects indicated that the sham group spent significantly more time in the correct than incorrect search zones, *F*(1, 17) = 50.44, *P* < 0.001, MSE = 11.07, whereas this difference was not significant for the hippocampal group, *F* < 1. In addition, significantly more time was spent by the sham group than the hippocampal group in the correct search zones, *F*(1, 17) = 15.03, *P* < 0.005, MSE = 8.97, while significantly less time was spent by the sham group than the hippocampal in the incorrect search zones, *F*(1, 17) = 7.24, *P* < 0.05, MSE = 7.75.

The test trials revealed that even when the difference between the long and short panels was considerable, the active training with the hippocampal group failed to result in any sort of preference for one wall over the other. This finding stands in stark contrast to the performance of the same group in Experiment 1, where it was able to make use of the black and white walls during active training to find the submerged platform. It thus appears that hippocampal lesions impair active spatial learning based on some stimuli, but not others.

Throughout the training of Phases 1 and 2, neither group displayed a significant preference for heading directly for a correct search zone on being released into the pool. In fact, on being released many rats from both groups eventually acquired a preference for heading in a particular direction, turn to the right, say, on being released in a corner. Such a strategy would ensure that the first platform they encountered would be correct on half of the trials, and ensure that neither measure of performance during active training would provide a satisfactory indication of the development of the discrimination.

Before we consider the theoretical significance of the present results, we describe one final experiment. The design was similar to that of Experiment 3, except that the four panels were of the same width, and contained black and white stripes rather than being entirely black. The stripes were vertical for one pair of opposing panels, and horizontal for the other pair, and the platforms were situated in the middle of panels with stripes of a given orientation. It is conceivable that some aspect of the methodology of Experiment 3, other than the differing widths of the panels, was responsible for the failure of the hippocampal group to identify where the platform could be found. If this is correct, then given the similarity of their designs, it would be expected that active training with the hippocampal group in Experiment 4 will be as impaired as for Experiment 3.

## EXPERIMENT 4

### Materials and Methods

#### Subjects

The subjects were 32 experimentally naïve rats from the same stock, and housed in the same manner as for Experiment 1. Prior to surgery they were assigned in equal numbers to the two groups. Following the completion of the histology, three rats from the hippocampal group were excluded from the experiment because their lesions were incomplete. At least 2 weeks after the surgery, the rats were used in an appetitive conditioning experiments that took place in standard conditioning chambers and for which they were reduced to 80% of their free-feeding weights. Following the completion of the experiment, they were allowed free access to food for two weeks before the start of the experiment.

#### Apparatus

The apparatus consisted of the same pool, walls, platforms, and landmarks as for Experiment 3. The only difference between the experiments was that the panels were all 100 cm wide, and 45 cm high and they all contained black and white stripes that were 5 cm wide. The stripes were horizontal for two of the panels, and vertical for the remaining two. Panels with stripes of the same orientation were attached to opposite walls of the square arena.

#### Surgery

The rats received either hippocampal lesions (*n* = 16) or sham lesions (*n* = 16) using the procedures described in Experiment 1.

#### Procedure

The experiment lasted for 11 sessions with four training trials in each of the first 10 sessions, and a single test trial in Session 11. The manner of training and testing was the same as for Experiment 3. Eight rats in the sham group, and eight in the hippocampal group were trained with the two platforms situated 15 cm from the center of the two panels with vertical stripes. The platforms were situated near the centers of the two panels with horizontal stripes for the remaining rats. Procedural details that have been omitted were the same as for Experiment 1.

## RESULTS

### Histology

**Figure 11:**
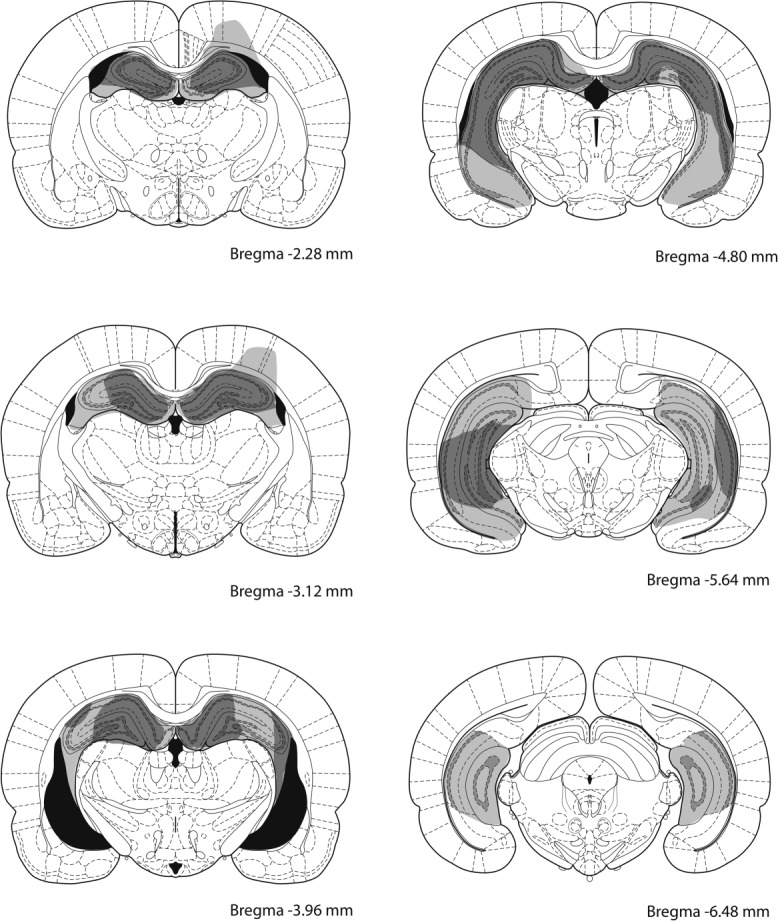
Schematic representation of ibotenic acid lesions of the hippocampus in Experiment 4. The largest and smallest extents of neuronal damage are represented in light grey and dark gray, respectively. Atlas plates are adapted from Paxinos and Watson (2005).

Figure 11 shows the extent of damage produced by the hippocampal lesions, which is similar to those lesions described in the previous experiments. Three rats were excluded from the behavioral analyses as the lesions were too small. The remaining 13 rats sustained damage ranging between 50 and 94% of the hippocampus, with more variation was again found in the ventral and posterior part.

### Behavior

**Figure 12:**
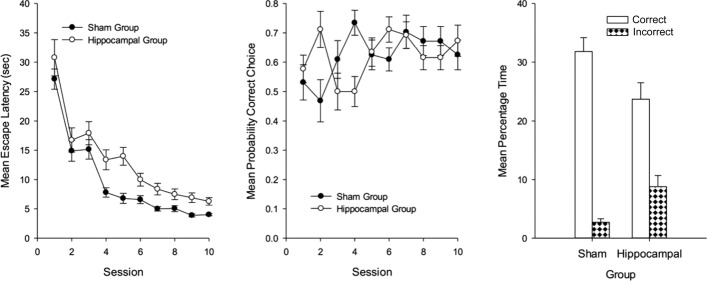
The mean escape latencies during training (left-hand panel), the mean probabilities of making a correct choice during training (center panel), and the mean percentages of time spent in the correct and incorrect search zones during the test trial (right-hand panel) for the two groups of Experiment 4. Error bars represent standard errors.

From the left-hand panel of Figure 12 it is evident that across the 10 sessions of training the mean escape latencies were longer for the hippocampal than the sham group, but in both cases performance improved as training progressed. A two-way ANOVA of individual mean escape latencies for each training session revealed a significant effect of session, *F*(9, 243) = 67.55, *P* < 0.001, MSE = 23.05, reflecting the gradual improvement across sessions, a significant effect of lesion, *F*(1, 27) = 18.98, *P* < 0.001, MSE = 47.73, but the interaction was not significant, *F* < 1.

The center panel of Figure 12 shows the mean probability of each group heading directly towards a correct search zone after being released into the pool across the 10 sessions of training. Both groups showed a slight improvement in this measure as training progressed, but there was rather little difference between them. A two-way ANOVA revealed no significant effect of session, *F*(9, 243) = 1.56, *P* > 0.10, MSE = 390.58, or no effect of lesion, *F* < 1. There was a significant Lesion × Session interaction, *F*(9, 243) = 3.00, *P* < 0.005, MSE = 390.58. Subsequent tests of simple main effects revealed a significant difference between the groups on Sessions 2 and 4, but in the opposite directions, *F*s(1, 27) > 5.81, *P*s < 0.05. One sample *t*-tests, based on individual mean probabilities of correct choice for the final five sessions combined, revealed a significant preference for heading directly for a correct search zone in both the sham, *t*(15)= 3.53, *P* < 0.005, and the hippocampal, *t*(12) = 4.50, *P* < 0.005, group.

The right-hand panel of Figure 12 shows the result from the single test trial at the end of the experiment. Both groups spent more time in the correct than incorrect search zones during the test, with the extent of this preference being greater for the sham than the hippocampal group. A two-way ANOVA revealed a significant effect of zone, *F*(1, 27) = 75.53, *P* < 0.001, MSE = 91.87, and a significant Lesion × Zone interaction, *F*(1, 27) = 7.89, *P* < 0.01, MSE = 91.87. The effect of lesion was not significant, *F* < 1. Subsequent tests of simple main effects showed that each group spent significantly more time in the correct than the incorrect search zones, *F*s(1, 27) > 15.67, *P*s < 0.001, MSE = 91.87. In addition the sham group spent significantly more time in the correct search zone, *F*(1, 27) = 4.87, *P* < 0.05, MSE = 97.24, and significantly less time in the incorrect search zone, *F*(1, 27) = 10.98, *P* < 0.005, MSE = 24.24, than the hippocampal group.

The principal difference between Experiments 3 and 4 is that in the former the position of the submerged platforms was indicated by the different lengths of the panels attached to the walls, whereas in the present study it was indicated by the orientation of the stripes within the panels. This difference led to the discrimination being rendered insoluble in rats with hippocampal lesions in Experiment 3, and the discrimination being disrupted, but not rendered insoluble in the current experiment. Taken together, the results from both experiments point forcefully to the conclusion that active spatial learning can be seriously affected by hippocampal lesions, but only in selected circumstances.

## GENERAL DISCUSSION

Four experiments investigated how passive and active spatial learning are affected by lesions of the hippocampus in rats. The first two experiments revealed that passive training of being placed at the goal was sufficient to allow the animals to identify its location with reference to the surrounding cues (see also Horne et al., 2012; Gilroy and Pearce, 2014), but this treatment was not effective in rats with lesions of the hippocampus. We argued that because passive training meant that rats had no experience of swimming in the square pool, these results indicate the hippocampus is critically important for what we refer to as spatial S-S* associations, where S represents cues provided by the walls of the square pool, and S* represents the goal of the submerged platform. Experiments 1 and 2, as well as Experiment 4, revealed that rats with hippocampal lesions are readily able to find a goal when they have experience of making their own way to it. These findings suggest that the hippocampus is less important for the formation of S-R than S-S* associations. The results from Experiment 3 indicate that the foregoing conclusion must be qualified in cases where S-R associations are based on information provided by the length of objects. In this instance, animals with hippocampal lesions were unable to identify where the goal was located, even when they had ample opportunity to find it while swimming freely in the pool.

A spatial S-S* association consists of a representation of some or all of the landmarks surrounding the goal (S), a representation of the goal itself (S*), and an association between these events. If it is accepted that hippocampal lesions disrupt the formation of such an association, it becomes important to identify precisely how the lesions are exerting their influence. One possibility is that they disrupt the associative learning process, and make it hard for one stimulus to be associated with another. The fact that pairing a conditioned and unconditioned stimulus for delay conditioning (e.g., Solomon and Moore, 1975; Solomon et al., 1986) or pairing two neutral stimuli for sensory preconditioning (e.g., Honey et al., 2014) is unaffected by damage to the hippocampus indicates that this possibility can be rejected. There is evidence that hippocampal damage can impair trace conditioning (e.g., Solomon et al., 1986; Bangasser et al., 2006), but since passive training in Experiments 1 and 2 allowed animals to experience simultaneously the landmarks and the goal, then it does not seem reasonable to regard this methodology as an instance of trace conditioning. It would thus seem that damage to the hippocampus in Experiments 1 and 2 was effective not because it resulted in a general impairment of the ability to acquire stimulus-stimulus associations. As an alternative, hippocampal lesions may have a specific impact on the acquisition of spatial S-S* associations.

A rather different explanation for at least some of our results can be based on the suggestion that the hippocampus is important for the formation of structural representations (Aggleton and Pearce, 2001), or conjunctive representations (Rudy and Sutherland, 1989), or representations of the context (Nadel and Wilner, 1980), or relational representations (Eichenbaum et al., 1992). Despite differing in detail, these accounts all assume that the hippocampus is important for representing the relationship between components of a configuration of stimuli. Any of these accounts would seem to provide a good explanation for the results of Experiment 1, because animals were required to differentiate between a corner where a black wall was to the left of a white wall, and the mirror image of this arrangement. If the hippocampus is important for acquiring information about the spatial relationship between the components of a pattern of stimulation, then damage to this region would be expected to impair the effectiveness of placement training in Experiment 1. The challenge for this explanation is to explain why the placement training was affected by hippocampal lesions in Experiment 2, when the platform was situated in a corner surrounded by two striped walls. On this occasion, there was no need to learn about structural information concerning the walls creating the correct corner, and yet the hippocampal group failed to benefit from the placement training. It thus appears that spatial S-S* associations involving even simple stimuli are difficult to acquire during placement training in rats with hippocampal lesions.

The effect of hippocampal lesions on passive spatial learning might be seen to be consistent with the claim that the hippocampus participates critically in the development of a cognitive map (O'Keefe and Nadel, 1978; Doeller et al., 2008), where passive training allows a normal rat to construct a map of the environment and the location of the goal within it. The inability of rats with hippocampal lesions to construct maps would then explain the disruptive effect of this manipulation on passive spatial learning. One problem for this account is that it is normally assumed that a map constitutes a global representation of the environment. As a test of this proposal, Gilroy and Pearce (2014) conducted a series of experiments similar to Experiments 1 and 2, with placement training being conducted in one corner of a square pool with distinctive walls. It was found that a change to the walls opposite the correct corner, by transposing them for example, had no impact on the effectiveness of placement training. If rats identified the correct corner by means of a global map of the entire arena, then such changes would be expected to disrupt the use of the map and make it difficult to identify where the platform was originally situated. The failure to find a disruptive influence of moving the distal wall suggests, therefore, that if rats acquire cognitive maps, they may be more localized than is normally considered to be the case.

To our knowledge, Experiments 1 and 2 are the first to investigate the impact of hippocampal lesions on spatial learning, when the animal is confined to the goal. Related studies have been conducted by White et al., but rats were able to move in a confined area as they learned where food was located. The apparatus consisted of an 8-arm radial maze, with arms that were 60 cm in length. Rats were first allowed to explore the maze, but with no food available. They were then confined to the outer halves of two arms where they could consume food in one arm but not the other. Following this training, rats were allowed to choose between the two arms. When the arms were adjacent to each other, then sham operated rats, but not rats with hippocampal lesions, exhibited a preference for the correct over the incorrect arm during the test trial (Chai and White, 2004, see also White and Gaskin, 2006). On the other hand, when the arms were on opposite sides of the maze, then disruption of hippocampal activity by fimbria-fornix lesions did not impair the ability of rats to identify the correct arm (White and McDonald, 1993). To explain this pattern of results, White (2008, pages 33-34) argued that a discrimination between two adjacent arms will be based on learning about the different relationships between the two arms and the same extramaze cues. Such stimulus-stimulus learning is said to depend upon the hippocampus. When the arms are opposite each other, then the rat is able to associate different extramaze cues with the presence and absence of food. Such stimulus-reinforcer associations are said to depend on the amygdala. One interpretation of these proposals is that the hippocampal lesions should not have exerted a disruptive influence in Experiments 1 and 2, because it was possible to identify the position of the platform by means of stimulus-reinforcer, or what we refer to as S-S* associations. However, the differences in methodology, apparatus, and reinforcer, make it very difficult to draw with confidence any conclusions from a comparison of the present results with those summarized by White (2008).

Turning now to the influence of hippocampal lesions on the effects of active training, each of the four experiments revealed a disruptive effect of the lesions. In Experiments 1, 2, and 4 this influence was minor, relative to the performance of the sham-operated control groups, and in each case the lesioned group revealed a strong preference for searching in the correct rather than incorrect search zones during the final test trial. In view of these results it is tempting to suggest that the capacity to form S-R associations is barely affected by damage to the hippocampus. Indeed, it is conceivable that this damage had no impact at all in these experiments on S-R learning, and the difference between the performance of the sham and hippocampal groups was due solely to the former taking advantage of the additional S-S* associations that would develop during training. When it comes to Experiment 3, then a very different conclusion needs to be drawn. The platform could be found by swimming to the middle of a long but not a short panel, and the discrimination was not solved by the hippocampal group. This result thus corroborates the claim by Pearce et al. (2004) that hippocampal lesions make it difficult for animals to discriminate between objects on the basis of their length. Once it is acknowledged that the hippocampus is involved in making judgement based on length, or distance, it is possible to understand the effect of lesions of this region on a variety of tasks where an animal must find its way to a hidden goal. Take, for example, the case where a rat has to swim to a submerged platform in a circular pool, with reference to landmarks outside the pool (Morris et al., 1982). If the position of the goal was defined by its relative distance from one or more landmarks, then any disruption of the ability to make judgements based on distance would make it difficult to find accurately the platform. Indeed, an impairment in the capacity to judge distance and length is likely to disrupt navigation in any task where the gaol is not situated immediately beside a salient landmark (e.g., Sutherland et al., 1983; Packard and McGaugh, 1992; Horne et al., 2010). Rather different support for the suggestion that the hippocampus is important for the accurate perception of length comes indirectly from the finding that hippocampal place cells fire maximally when the individual is a certain distance from the cues that control firing rate (e.g., O'Keefe and Dostrovsky, 1971; Muller et al., 1987; O'Keefe and Burgess, 1996).

O'Keefe and Nadel (1978) drew a distinction between taxon learning, in which the location of a goal is indicated by landmarks that are close to it, and locale learning, in which the location of a goal is indicated by its relationship with an array of landmarks that are some distance from it. They further suggested that the hippocampus plays an important role in locale, but not taxon learning. For related views see Packard and McGaugh (1992,1996), and Doeller, King and Burgess (2008). These proposals have gained considerable support from the repeated finding that damage to the hippocampus will impair the ability of animals to find a goal when they must rely on distal cues, but not when they can make use of local cues provided by a landmark at or near the goal (e.g., Morris et al., 1986; Pearce et al., 1998; Devan and White, 1999). The results from the present experiments do not fit comfortably with these proposals of O'Keefe and Nadel (1978). In particular, in all four experiments the escape platform was situated near a distinctive landmark that could, presumably, be used to indicate the position of the platform by means of taxon learning. On the basis of the proposals of O'Keefe and Nadel (1978), therefore, the hippocampal lesions should not have disrupted the effects of passive training in Experiments 1 and 2, or the effects of active training in Experiment 3. The clear findings to the contrary thus suggests that the hippocampus occasionally plays an important role in taxon learning, and there is a need to understand what this role might be. It is possible that by seeking to establish a detailed understanding of the conditions under which lesions of the hippocampus influence spatial behavior we shall gain important new insights into the role of this region. With this end in mind, the present experiments have shown that the hippocampus plays an essential role in passive spatial learning. It appears to play less of an important role in active spatial learning, except when reference must be made to information based on distance.
